# Supplementary material for: Liquid Chromatography ICP-MS to Assess the Stability of 175Lu- and natGa-Based Tumor-Targeting Agents towards the Development of 177Lu- and 68Ga-Labeled Radiopharmaceuticals
Source: Pharmaceutics. 2024 Feb 21;16(3):299. doi: 10.3390/pharmaceutics16030299 (PMC10974936; doi:10.3390/pharmaceutics16030299)
Supplement: Supplementary file 1 [file pharmaceutics-16-00299-s001.zip › pharmaceutics-2772426-supplementary.pdf]

pharmaceutics

Supplementary Materials: Liquid Chromatography ICP-MS to Assess the Stability of ^{175}Lu- and ^{nat}Ga-Based Tumor-Targeting Agents towards the Development of ^{177}Lu- and ^{68}Ga-Labeled Radiopharmaceuticals

Rahel H. Wallimann, Heloïse Hensinger, Cristina Müller, Roger Schibli, Rainer Kneuer and Patrick Schindler

S1. Synthesis of biologics metal conjugates

Purpose: Reference biologics metal conjugates were synthesized to demonstrate that size exclusion chromatography (SEC) coupled to inductively coupled plasma mass spectrometry (ICP-MS) could serve as a surrogate method to radio-HPLC.

Experimental procedure: Bioconjugation of the anti-PSMA-sdAb and anti-PSMA-mAb with the bifunctional chelators DOTAGA and NODAGA, respectively, was done as previously described in the literature [1-4]. Metal labeling of the biologics conjugates with the stable isotopes of lutetium and gallium is described in the main manuscript.

Results: The sdAb and mAb metal conjugates were successfully synthesized, as described in the main article. The identity of the final metal conjugates was confirmed by analytical SEC and LC-MS (Table S1).

Table S1. Analytical data of synthesized ^{175}Lu- and ^{nat}Ga-labeled anti-PSMA-sdAb conjugates and anti-PSMA-mAb conjugates.

Conjugate	Theor. Mass[a] [Da]	Exp. Mass[b] [Da]	t_r[c] [min]	Chemical purity[d] [%]
[^{175}Lu]Lu-DOTAGA-cys-mal-anti-PSMA-sdAb	13'077.2	13'078.0	6.10	>99%
[^{nat}Ga]Ga-DOTAGA-cys-mal-anti-PSMA-sdAb	12'972.6	12'972.9	6.08	>99%
[^{nat}Ga]Ga-NODAGA-cys-mal-anti-PSMA-sdAb	12'871.1	12'871.7	6.03	>98%
[^{175}Lu]Lu-DOTAGA-LPETGG-anti-PSMA-sdAb	13'458.7	13'459.0	6.02	>99%
[^{175}Lu]Lu-DOTAGA-cys-mal-anti-PSMA-mAb	LC: 23’421.5<br>HC: 48’618.0<br>(unconjug.)	LC: 23’422.0<br>HC: 49’407.0 (DAR 1)<br>HC: 50’179 (DAR 2)	8.60	>99%
[^{nat}Ga]Ga-NODAGA-cys-mal-anti-PSMA-mAb	LC: 23’421.5<br>HC: 48’357.0<br>(unconjug.)	LC: 23’422.0<br>HC: 49’200.0 (DAR 1)	8.60	>99%
[^{175}Lu]Lu-*p*-NCS-bz-DOTAGA-anti-PSMA-mAb	LC: 23'517.8<br>HC: 49'441.1<br>(unconjug.)	LC: 23'519.0<br>LC: 24'314.0 (DAR 1)<br>LC: 25'109.0 (DAR 2)<br>HC: 49'440.0	8.56	96%
[^{nat}Ga]Ga-*p*-NCS-bz-NODAGA-anti-PSMA-mAb	LC: 23'517.8<br>HC: 48'644.7<br>(unconjug.)	LC: 23'519.0<br>HC: 48'645.0<br>HC: 49'232.0 (DAR 1)	8.66	91%
[^{165}Ho]Ho-DOTA-BSA	66'421.6<br>(unconjug.)	67'135.0 (DAR 1)<br>67'848.6 (DAR 2)<br>68'562.1 (DAR 3)<br>69'275.8 (DAR 4)<br>69'988.7 (DAR 5)	3.97	>99%

[a] Theoretical average molecular mass; [b] Measured molecular mass (LC-MS) with the drug antibody ratio (DAR); [c] retention time of the conjugate on analytical size exclusion chromatography (SEC). The column utilized for the analysis of the sdAb conjugates and the [^{165}Ho]Ho-DOTA-BSA was a 5/150 GL Superdex 75 pg column, with phosphate buffered saline (PBS), pH 7.4, as mobile phase and an isocratic flow of 0.3 mL/min over 18 min. The column utilized for the analysis of the mAb conjugates was a XBridge BEH SEC column, 200 Å, 3.5 µm, 7.8x150 mm, with PBS, pH 7.4, as mobile phase and an isocratic flow of 0.4 mL/min over 18 min; [d] Determined by analytical SEC, λ = 280 nm and λ = 214 nm

S2. Reversed phase chromatography to analyze *in vitro* serum stability assays of [^{175}Lu]Lu-PSMA-617 and [^{nat}Ga]Ga-PSMA-11

Purpose: To prove that the elaborated reversed phase chromatography (RPC)-ICP-MS can be used to detect the potential loss of metal ions from the metal-chelator complex, mixtures of metal-labeled peptidomimetics and DTPA-/EDTA-complexed metal ions were analyzed. To define the linear range and to confirm the robustness of the RPC-ICP-MS technique, calibration curves for the metal-labeled peptidomimetics and for metal ions were prepared and analyzed.

Experimental Procedure: The preparation of the mixtures of metal-containing relevant species and of the calibration curves is described in the main article.

Results: RPC was successfully coupled to ICP-MS to allow the assessment of the chemical purity of ^{175}Lu- and ^{nat}Ga-labeled peptidomimetics after metal-labeling and the analysis of serum stability assays. The results are described in the main article. Separation of the metal ions from the peptidomimetics was achieved (Figure S1).

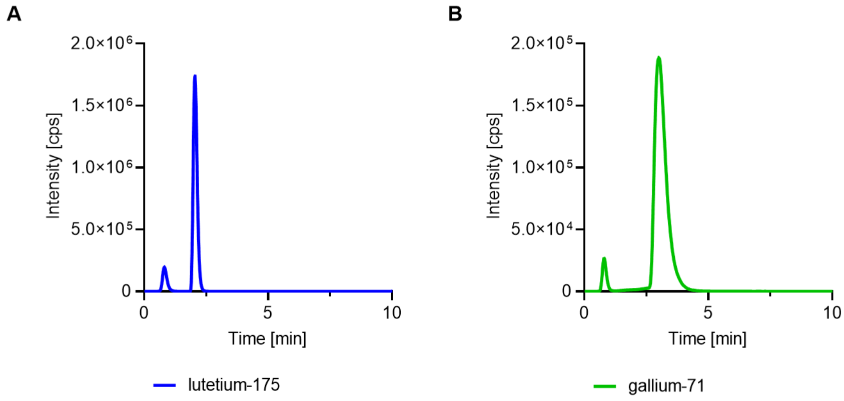

Figure S1. Representative chromatograms of relevant ^{175}Lu- and ^{nat}Ga-labeled species. (**A**) [^{175}Lu]Lu-PSMA-617 (t_R = 2.1 min) mixed with lutetium-175 ions complexed by DTPA (t_R = 0.6 min); (**B**) [^{nat}Ga]Ga-PSMA-11 (t_R = 1.8 min) mixed with gallium-69/gallium-71 ions complexed by EDTA (t_R = 0.8 min).

The linear range determined for [^{175}Lu]Lu-PSMA-617 was from 10 fmol to 12 pmol injected ligand, for lutetium-175 ions complexed by DTPA from 0.2 fmol to 286 fmol injected metal ions, for [^{nat}Ga]Ga-PSMA-11 from 16 fmol to 25 pmol injected ligand, and for gallium67/gallium-71 ions complexed by EDTA from 10 fmol to 5.6 pmol injected metal ions (Figure S2).

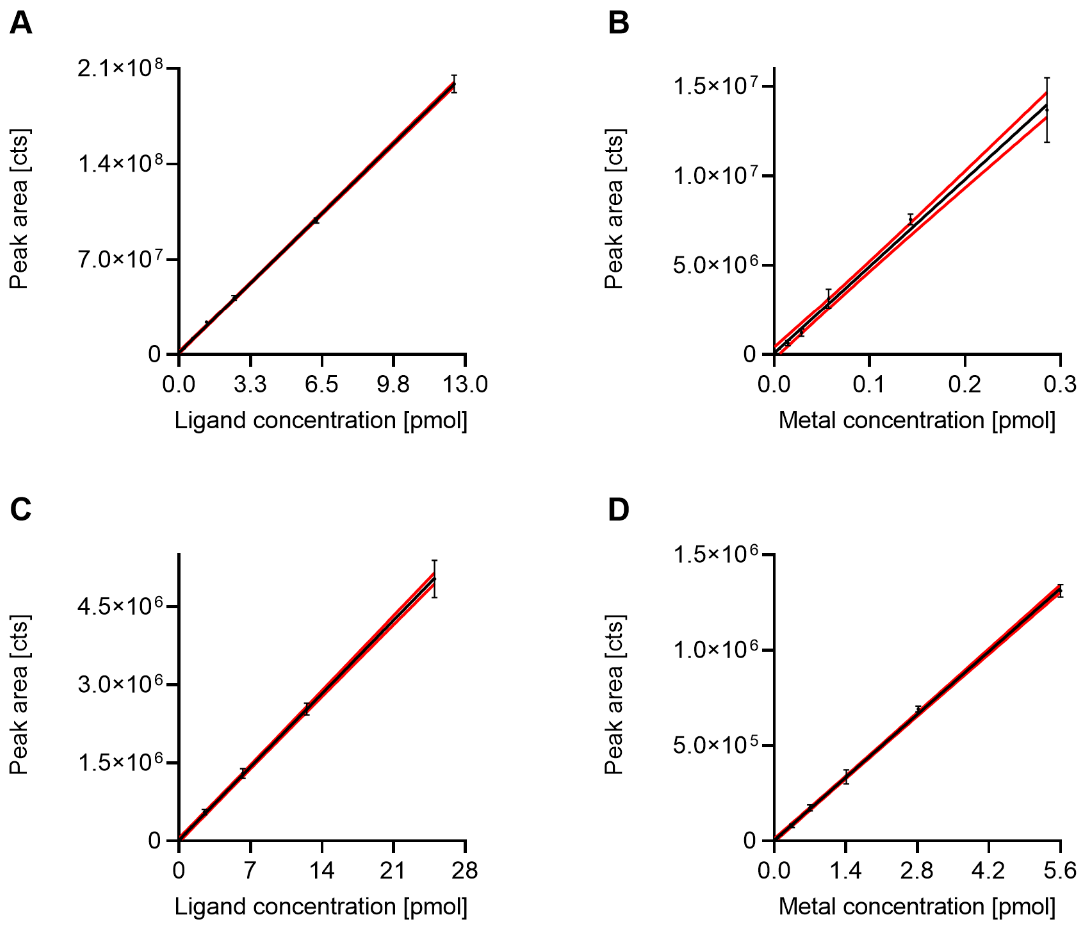

Figure S2. Calibration curves of ^{175}Lu- and ^{nat}Ga-labeled peptidomimetics and metal ions complexed by DTPA or EDTA. The detected peak areas were plotted against the molar amount of injected metal and are given as average ± standard deviation (n = 3). Simple linear regression analysis was applied. The best-fit line and the 95% confidence bands (red lines) of the best-fit line were plotted using GraphPad Prism software (version 9). (**A**) [^{175}Lu]Lu-PSMA-617 (R^2 = 0.9982); (**B**) lutetium-175 complexed by DTPA (R^2 = 0.9822); (**C**) [^{nat}Ga]Ga-PSMA-11 (R^2 = 0.9957); (**D**) gallium-69/gallium-71 complexed by EDTA (R^2 = 0.9981).

S3. Size exclusion chromatography ICP-MS to analyze *in vitro* serum stability assays of ^{175}Lu- and ^{nat}Ga-labeled single domain antibody conjugates

Purpose: For peak identification of metal-labeled species obtained in serum stability assays, mixtures of reference standards were analyzed by SEC-ICP-MS. To identify the linear range and to confirm the robustness of the elaborated SEC-ICP-MS methods, calibration curves of metal conjugates and metal ions were prepared and analyzed.

Experimental procedure: The preparation of the mixtures of metal-containing relevant species and the calibration curves is described in the main article.

Results: SEC was successfully coupled to ICP-MS to allow the detection of relevant ^{175}Lu- and ^{nat}Ga-labeled species obtained by *in vitro* serum stability assays of sdAb metal conjugates (Figure S3).

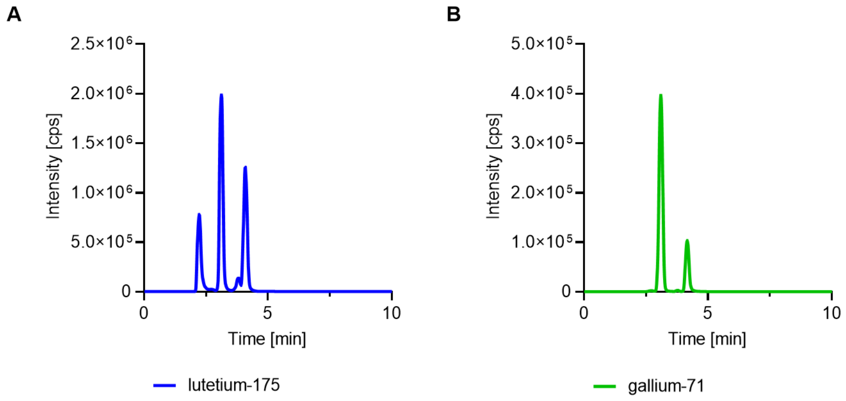

Figure S3. Representative chromatograms of relevant ^{175}Lu- and ^{nat}Ga-labeled species to show separation of the metal conjugates on a protein BEH SEC column (BEH SEC, 125 Å, 1.7 µm, 4.6x150 mm). Ammonium acetate (20 mM, pH 5.0) was used as mobile phase with an isocratic flow of 0.4 mL/min. (**A**) [^{175}Lu]Lu-DOTAGA-cys-mal-anti-PSMA-sdAb (t_R = 3.1 min) mixed with [^{175}Lu]Lu-DOTAGA-cys-mal-anti-PSMA-mAb (t_R = 2.2 min), [^{175}Lu]Lu-EDTA (t_R = 3.8 min), and [^{175}Lu]Lu-DOTAGA-mal (t_R = 4.1 min); (**B**) [^{nat}Ga]Ga-DOTAGA-cys-mal-anti-PSMA-sdAb (t_R = 3.1 min) mixed with [^{nat}Ga]Ga-EDTA (t_R = 4.1 min).

The linear range determined for [^{175}Lu]Lu-DOTAGA-cys-mal-anti-PSMA-sdAb was from 0.44 fmol to 1.4 pmol injected metal conjugate, for lutetium-175 ions complexed by DTPA from 0.68 fmol to 0.43 pmol injected metal ions, for [^{nat}Ga]Ga-DOTAGA-cys-mal-anti-PSMA-sdAb from 14 fmol to 4.9 pmol injected metal conjugate, and for gallium67/gallium-71 ions complexed by EDTA from 70 fmol to 1.4 pmol injected metal ions (Figure S4).

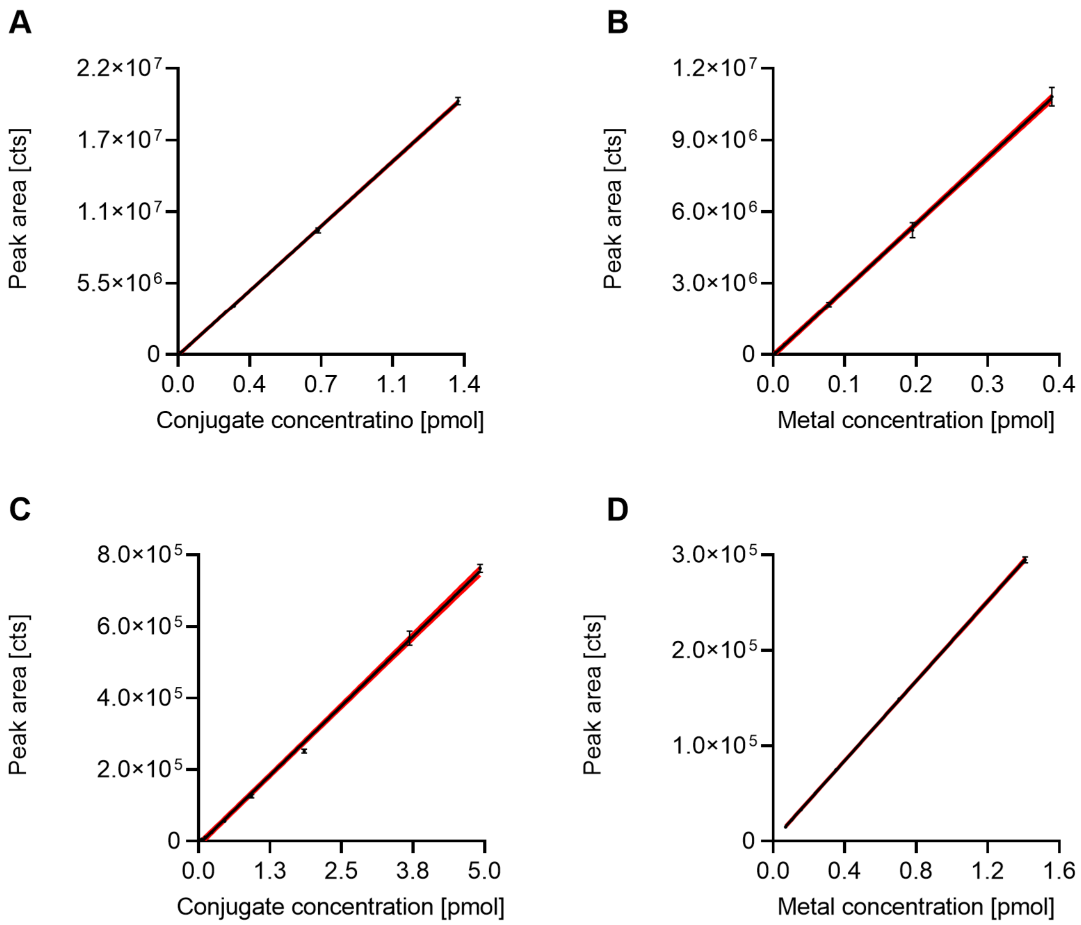

Figure S4. Calibration curves of ^{175}Lu- and ^{nat}Ga-labeled sdAb-conjugates and metal ions complexed by DTPA or EDTA. The detected peak areas were plotted against the molar amount of injected metal and are given as average ± standard deviation (n = 3). Simple linear regression analysis was applied. The best-fit line and the 95% confidence bands (red lines) of the best-fit line were plotted using GraphPad Prism software (version 9). (**A**) [^{175}Lu]Lu-DOTAGA-cys-mal-anti-PSMA-sdAb (R^2 = 0.9996); (**B**) lutetium-175 complexed by DTPA (R^2 = 0.9972); (**C**) [^{nat}Ga]Ga-DOTAGA-cys-mal-anti-PSMA-sdAb (R^2 = 0.9978); (**D**) gallium-69/gallium-71 complexed by EDTA (R^2 = 0.9998).

S4. Analysis of *in vitro* serum stability assays of metal-labeled antibody conjugates by SEC-ICP-MS

Purpose: For the analysis of *in vitro* serum stability assays of metal-labeled antibody conjugates, an AQUITY UPLC protein SEC column (Premier Protein SEC, 250 Å, 1.7 µm, 4.6x150 mm, Waters, Milford, MA, USA) was used. The resolution of this column allowed the quantification of the metal-labeled fraction co-eluting with serum proteins.

Experimental procedure: Mixtures of metal-labeled standards and EDTA-complexed [^{175}Lu]Lu^{3+} and [^{nat}Ga]Ga^{3+} were prepared and analyzed according to the methods described in the main article. The standards for the calibration curves were prepared as described in the main article, using mouse serum diluted in a ratio of 1:9 (*v*/*v*) in a mixture of 0.1% TFA (*v*/*v*) in H_2O, EDTA (17 µM), and [^{165}Ho]Ho-DOTA-BSA (9 nM).

Results: Sufficient peak separation of the metal conjugate from relevant metal-labeled species to allow the analysis of *in vitro* serum stability by SEC-ICP-MS was achieved (Figure S5).

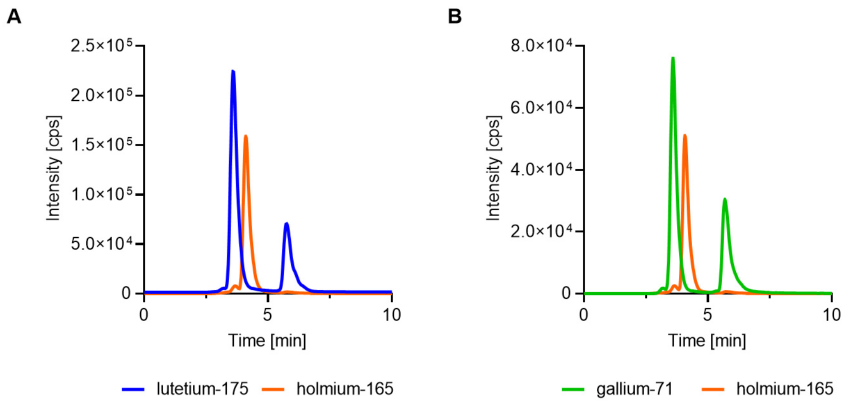

Figure S5. Representative chromatograms of relevant ^{175}Lu- and ^{nat}Ga-labeled species using a Premier Protein SEC column. 50% ammonium acetate (200 mM, pH 7.0) was used as a mobile phase with an isocratic flow of 0.3 mL/min. (**A**) [^{175}Lu]Lu-DOTAGA-cys-mal-anti-PSMA-mAb (t_R = 3.6 min) mixed with [^{165}Ho]Ho-DOTA-BSA (t_R = 4.1 min), and [^{175}Lu]Lu-EDTA (t_R = 5.8 min); (**B**) [^{nat}Ga]Ga-NODAGA-cys-mal-anti-PSMA-mAb (t_R = 3.6 min) mixed with [^{165}Ho]Ho-DOTA-BSA (t_R = 4.1 min) and [^{nat}Ga]Ga-EDTA (t_R = 5.7 min).

For [^{175}Lu]Lu-DOTAGA-cys-mal-anti-PSMA-mAb the linear range was defined between 1.8 fmol and 1.8 pmol injected lutetium-175, whereas for lutetium-175 complexed by EDTA, it was defined between 6.9 fmol and 3.4 pmol. For [^{nat}Ga]Ga-NODAGA-cys-mal-anti-PSMA-mAb and gallium-69/gallium-71 complexed by EDTA, the linear range was determined between 1.3 pmol and 104 pmol, and between 51 fmol and 6.5 pmol, respectively (Figure S6).

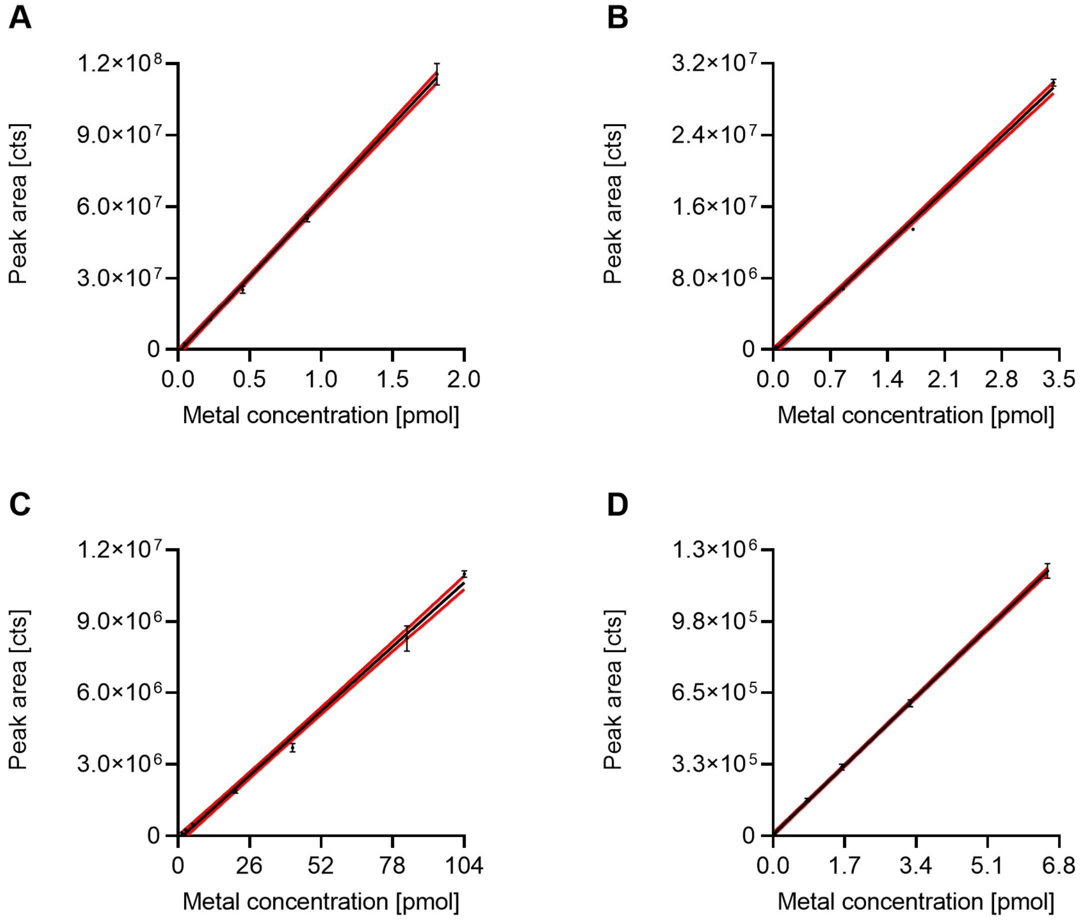

Figure S6. Calibration curves of ^{175}Lu- and ^{nat}Ga-labeled mAb-conjugates and metal ions complexed by EDTA. The detected peak areas were plotted against the molar amount of injected metal and are given as average ± standard deviation (n = 3). Simple linear regression analysis was applied. The best-fit line and the 95% confidence bands (red lines) of the best-fit line were plotted using GraphPad Prism software (version 9). (**A**) [^{175}Lu]Lu-DOTAGA-cys-mal-anti-PSMA-mAb (R^2 = 0.9973); (**B**) [^{175}Lu]Lu-EDTA (R^2 = 0.9975); (**C**) [^{nat}Ga]Ga-NODAGA-cys-mal-anti-PSMA-mAb (R^2 = 0.9942); (**D**) [^{nat}Ga]Ga-EDTA (R^2 = 0.9990).

S5. *In vitro* serum stability of [^{175}Lu]Lu-PSMA-617 and [^{nat}Ga]Ga-PSMA-11

Purpose: The aim of the experiment was to demonstrate that the elaborated RPC-ICP-MS technique could be used for the analysis of *in vitro* serum stability experiments of ^{175}Lu- and ^{nat}Ga-labeled peptidomimetics.

Experimental procedure: [^{175}Lu]Lu-PSMA-617 and [^{nat}Ga]Ga-PSMA-11 were incubated in mouse serum (biowest, sterile filtered, Bradenton, FL, USA) at 37 °C at a molar concentration of 5 µM. Aliquots were taken at the time points 0 h, 1 h, 2 h, 4 h, 6 h, and 24 h and stored at -80 °C to stop the reaction. To allow analysis of the serum stability samples by RPC-ICP-MS, precipitation of proteins present in the mouse serum and extraction of the ligands was required. Extraction solvents with either varying ratios of ACN:H_2O, including pure ACN, ACN:H_2O 9:1 (*v/v*), ACN:H_2O 8:2 (*v/v*), ACN:H_2O 7:3 (*v/v*), or MeOH, and a mixture of ACN:MeOH:H_2O 1:1:1 (*v/v*) + 0.1% formic acid were investigated. The highest recovery of the ligands (>90%) was achieved with ACN:H_2O 7:3 (*v/v*). For the extraction of [^{175}Lu]Lu-PSMA-617 and [^{nat}Ga]Ga-PSMA-11 from the mouse serum, a six time excess of a mixture of ACN:H_2O 7:3 (*v/v*) containing [^{165}Ho]Ho-PSMA-617 (5 µM and 10 nM, respectively) was added to the aliquots of the serum stability samples. [^{165}Ho]Ho-PSMA-617 was used as internal standard. The probes were vortexed for 30 sec and subsequently centrifuged for 20 min using a bench mini-centrifuge (Mini Star from VWR, Lutterworth, UK). The obtained supernatant was five times diluted with H_2O containing DPTA (254 µM) to allow subsequent analysis by the elaborated techniques for RPC-ICP-MS.

Results: High chemical stability of >99% for both, [^{175}Lu]Lu-PSMA-617 and [^{nat}Ga]Ga-PSMA-11, was confirmed by RPC-ICP-MS. The obtained values were compared to the stability data of the corresponding radiopharmaceuticals [^{177}Lu]Lu-PSMA-617 (24 h) [5] and [^{68}Ga]Ga-PSMA-11 (1 h) [6] reported in the literature (Table S2). Due to the short half-live of gallium-68 (68 min), the stability of [^{68}Ga]Ga-PSMA-11 was determined after 1 h incubation.

Table S2. In vitro serum stability of [^{175}Lu]Lu-/[^{177}Lu]Lu-PSMA-617 and [^{nat}Ga]Ga-/[^{68}Ga]Ga-PSMA-11.

Ligand	In vitro serum stability of metal conjugate	In vitro serum stability of radioligand
Lu-PSMA-617	>99%	>99%*
Ga-PSMA-11	>99%	>95% (1 h)†

* Data obtained for [^{177}Lu]Lu-PSMA-617 was previously published by Chakraborty *et al.* Cancer Biother Radiopharm 2021 [5] and was added for comparison.

†Data obtained for [^{68}Ga]Ga-PSMA-11 was previously published by Fuscaldi *et al.* Pharmaceuticals 2021 [6] and was added for comparison.

References

1. 1. Chatalic, K.L.; Veldhoven-Zweistra, J.; Bolkestein, M.; Hoeben, S.; Koning, G.A.; Boerman, O.C.; de Jong, M.; van Weerden, W.M. A novel ^{111}In-labeled anti–prostate-specific membrane antigen nanobody for targeted SPECT/CT imaging of prostate cancer. *J Nucl Med* **2015**, *56*, 1094-1099.
2. 2. Massa, S.; Vikani, N.; Betti, C.; Ballet, S.; Vanderhaegen, S.; Steyaert, J.; Descamps, B.; Vanhove, C.; Bunschoten, A.; van Leeuwen, F.W. Sortase A-mediated site-specific labeling of camelid single-domain antibody-fragments: a versatile strategy for multiple molecular imaging modalities. *Contrast Media Mol Imaging* **2016**, *11*, 328-339.
3. 3. Coumans, R.G.; Ariaans, G.J.; Spijker, H.J.; Renart Verkerk, P.; Beusker, P.H.; Kokke, B.P.; Schouten, J.; Blomenröhr, M.; van der Lee, M.M.; Groothuis, P.G. A Platform for the Generation of Site-Specific Antibody–Drug Conjugates That Allows for Selective Reduction of Engineered Cysteines. *Bioconjug Chem* **2020**, *31*, 2136-2146.
4. 4. Le Bihan, T.; Navarro, A.-S.; Le Bris, N.; Le Saec, P.; Gouard, S.; Haddad, F.; Gestin, J.-F.; Cherel, M.; Faivre-Chauvet, A.; Tripier, R. Synthesis of C-functionalized TE1PA and comparison with its analogues. An example of bioconjugation on 9E7. 4 mAb for multiple myeloma ^{64}Cu-PET imaging. *Org Biomol Chem* **2018**, *16*, 4261-4271.
5. 5. Chakraborty, A.; Mitra, A.; Tawate, M.; Sahoo, S.; Lad, S.; Rakshit, S.; Gaikwad, S.; Basu, S.; Shimpi, H.; Banerjee, S. Therapeutic multidose preparation of a ready-to-use ^{177}Lu-PSMA-617 using carrier added lutetium-177 in a hospital radiopharmacy and its clinical efficacy. *Cancer Biother Radiopharm* **2021**, *36*, 682-692.

6. 6. Fuscaldi, L.L.; Sobral, D.V.; Durante, A.C.R.; Mendonça, F.F.; Miranda, A.C.C.; da Cunha, M.L.; Malavolta, L.; Mejia, J.; de Barboza, M.F. Standardization of the [^{68}Ga] Ga-PSMA-11 radiolabeling protocol in an automatic synthesis module: assessments for PET imaging of prostate cancer. *Pharmaceuticals* **2021**, *14*, 385.

Disclaimer/Publisher's Note: The statements, opinions and data contained in all publications are solely those of the individual author(s) and contributor(s) and not of MDPI and/or the editor(s). MDPI and/or the editor(s) disclaim responsibility for any injury to people or property resulting from any ideas, methods, instructions or products referred to in the content.
